# A multi-country mixed-method study identifying the association between perceived ethical work climate and problems among critical care nurses

**DOI:** 10.1186/s12912-024-01861-9

**Published:** 2024-03-27

**Authors:** Fatma Refaat Ahmed, Nabeel Al-Yateem, Farha Hisham Hijji, Ghadeer Al Dweik, Mohammad Alkawaldeh, Muhammad Arsyad Subu, Jacqueline Maria Dias, Mohannad Eid Aburuz, Sally Mohammed Farghaly

**Affiliations:** 1 Department of Nursing, College of Health Sciences, University of Sharjah, Sharjah, UAE; 2 Critical Care and Emergency Nursing Department, Faculty of Nursing, Alexandria University, Alexandria, Egypt; 3 Nurse Consultant, Emirates Health Services, Sharjah, UAE; 4 Clinical Nursing Department, Faculty of Nursing, Applied Science Private University, Amman, Jordan; 5 Nursing Management and Education Department, College of Nursing, Princess Nourah bint Abdulrahman University, Riyadh, Saudi Arabia; 6 Nursing Administration Department, Faculty of Nursing, Alexandria University, Alexandria, Egypt

**Keywords:** Ethical problems, Ethical climate, Infectious diseases, ICUs, Nurses

## Abstract

**Background:**

Given the grave ethical tension and dilemmas posed continuously which are aggravated in the intensive care unit context and its related caregiving provision, combined with their impact on critical care nurses’ job satisfaction and work-related risks, exploring and analyzing these tensions and conflicts is crucial. This study was conducted to examine the relationship between perceived ethical work climate and problems among critical care nurses in addition to exploring their perspectives on the ethical work climates while caring for patients with infectious diseases.

**Design and method:**

A mixed-method research design was used to conduct this study among 635 participants, comprising 170 from Egypt, 144 from Jordan, 161 from Saudi Arabia, and 160 from the United Arab Emirates. Online or paper-based survey forms were distributed to all eligible critical care nurses who agreed to take part in the study. The survey contained both quantitative and qualitative data that were analyzed separately and integrated during the discussion. The study was reported following the STROBE guidelines.

**Findings:**

The overall ethical work climate was fairly good and was significantly associated with ICU nurses’ personal and professional characteristics. The findings also identified three main themes: (1) an ethical sense of failure, (2) environmental condemnation, and (3) an instant action plan for resolving ethical conflicts.

**Conclusions:**

ICU nurses perceived that ICU ethical climate was fairly good. The results indicated that ICU nurses generally had a relatively fair perception of the ethical work climate, with implications for addressing ethical issues and conflicts in various settings.

**Impact:**

Mentorship and/or close supervision concerning ethical resilience, consultation, and decision-making is crucial in the ICU milieu. Metacognitive strategies to reinforce problem-solving and decision-making ICU nurses’ skills could help them overcome the different ethical challenges. Adequate resources, teamwork, and organizational support are promising tactics to improve ICU nurses’ ethical skills.

**Trial registration:**

Not applicable.

**Supplementary Information:**

The online version contains supplementary material available at 10.1186/s12912-024-01861-9.

## Introduction

Intensive care units (ICUs) are one of the hospital’s most stressful and dynamic areas, in which nurses are subjected to various ethical problems [1]. These include issues related to informed consent [2], breaching of confidentiality [3], protection of patient rights [4], the provision of some unduly aggressive treatments [5], and failure to conduct end-of-life care measures [6], all of which could contribute to the development of ethical problems among critical care nurses, and affect their moral sensitivity [7]. The intensive nature of patients’ diseases, for example, infectious diseases and required treatment in the ICU could add more ethical burdens on nurses, especially with a low nurse-patient ratio, high workloads, and limited nursing time [8].

An ethical work climate is an important variable affecting nurses’ behavior, practice, and competence [9]. An ethical climate can be assessed by perceptions of how ethical issues in their work environment are handled [10]. Different ethical climates can be categorized according to whether they emphasize maximizing one’s own self-interests, maximizing the interests of others, or adhering to universal principles [11]. Victor and Cullen [11], states in their framework that deontology (upholding moral principles), benevolence (the welfare of others), and egoism (self-serving) serve as the foundation for moral decision-making. They suggest five different kinds of ethical climates based on these tenets: instrumental, rules and regulations, caring, independence, and rules. Olson’s conceptual framework of hospital ethical climate also posits that the ethical climate affects nurses’ level of performance, and a poor climate can reduce their work satisfaction, exacerbating stress and burnout, and increasing health system costs [12]. In the context of the Middle East, a study conducted in Iran by Tehranineshat [9] confirmed this hypothesis, reporting that the ethical work climate was associated with nurses’ professional quality of life. In Saudi Arabia, a study done by Almutairi et al., 2015 [7] reported that healthcare providers including nurses frequently deal with difficult ethical problems and practical decisions in their everyday work in healthcare settings. This would affect their wellbeing and increase their likelihood to moral distress.

Related to this dimension, an unpredictable work environment, ethical conflicts, and reluctant professional roles can lead to decreased quality of patient care, poor organizational dedication, moral distress, job dissatisfaction and burnout, and increased turnover rate [13–15]. Given the grave ethical tensions and dilemmas continuously arising in ICU contexts and related caregiving provision, combined with their impact on ICU nurses’ job satisfaction and work-related risks exploring and analyzing these tensions and conflicts is crucial in different contexts and cultures, for example, conflicts related to informed consent, confidentiality, and justice in the distribution of healthcare resources [4, 5, 16].

It is also valuable to evaluate the relationships between ICU nurses perceived ethical work climate, their personal and professional characteristics considering a deep understanding to their own perceptions. Consequently, this study focuses on perceived ethical work climate and risk among critical care nurses while caring for patients with infectious diseases as the first step to control such conflicts. The severity of the infectious diseases has dramatically exacerbated the ICU nurses’ ethical tension and challenge their responsibilities of protecting themselves, their families, community and from infection [13].

Ethical problems among ICU nurses might depend largely on professional and cultural background required in patient care [17]. However, despite many studies investigating professional competencies worldwide [1, 8, 18], the cultural background of nurses has largely been ignored, despite it being acknowledged to exert an important effect on their responses to ethical dilemmas. Accordingly, studying these variables among ICU nurses who are working in the Arab healthcare settings is pivotal.

Merriam-Webster [19] defines the concept of “culture” as “the set of values, conventions, or social practices associated with a particular field, activity, or societal characteristic”. Arab countries draw shared cultural beliefs and values, particularly those of the Middle Eastern region. Due to their shared history, religion, ethnic identity, language, and nationality, Arabs have a common set of traditions, behaviors, values, and belief sets. ICU nurses targeted in this study are from four different Arab countries where most of them are Arabs while others are expatriates. However, even expatriate nurses are (or ought to be) expected to exert cultural competence in treating majoritarian Arab service user populations. Therefore, to draw a meaningful and relevant conclusion about the ethical work climate in the Arabic countries, it is essential to study these variables rather than depending on similar studies done elsewhere in the other countries when developing evidence-based guidelines for fostering appropriate ethical caring ICU environments.

## Methods

### Aim and objectives

The aim of this study is to explore the ethical work climate in the ICUs within Arabic healthcare settings. The study included two phases: quantitative and qualitative one. The study examines the relationship between perceived ethical work climate and problems among critical care nurses. Through the qualitative component in the study, a deeper understanding of the nurses’ perspectives on the ethical work climates in their setting is achieved. This study aim is achieved by addressing four main research objectives, namely to:

1. Describe the frequency of proposed ethical problems in ICU.
2. Measure the ethical work climate as perceived by ICU nurses.
3. Determine the association between nurses’ personal and professional characteristics, perceived problems, and ethical work climate.
4. Understand the nurses’ perspectives on the ethical conflicts in ICU and how should be resolved.

### Study design

A mixed-method research design [20] was used to conduct this study. Previous studies in literature mainly adopted single method approaches to study this issue, which might be unsuitable to explore all aspects of nurses’ experience with ethical problems. This study adopted the triangulation of data, which is necessary to elicit all dimensions of participants’ experiences.

### Study settings

This study was conducted in various adult ICUs across four Arab countries, namely Egypt, Jordan, the Kingdom of Saudi Arabia (KSA), and the United Arab Emirates (UAE). The sample included two governmental facilities from UAE, two governmental hospitals in KSA, two university hospitals located in Alexandria, Egypt, and one governmental hospital in Amman, Jordan. The studied hospitals’ ICUs receive patients from the general public with different disorders in acute stages of illness including infectious diseases.

### Study participants

Nurses working in the ICUs for more than six months were recruited conveniently to take part in the study. A pilot study was conducted with 10% of the study sample to examine the feasibility of the study; nurses who participated in the pilot study were excluded from the final study sample. Following the pilot, 928 participants invited to participate, 635 agreed to participate and were ultimately included in this study (170 from Egypt, 144 from Jordan, 161 from KSA, and 160 from UAE). Only 89 participants agreed to engage into the qualitative phase of the study.

### Measurements

#### Quantitative component (objectives 1, 2, and 3)

Data were collected concurrently from the four Arab countries. An online survey (described below) was distributed to all eligible critical care nurses who were agreed to fill it out in Jordan, KSA, and UAE, while paper-based forms were used in Egypt. Questions were presented in English in each of the four countries. Most of nurses working in UAE [21] and Saudi Arabia [22] are expatriates who do not speak Arabic as a first language. In Egypt and Jordan all of the participants happened to be native citizens, who are fluent in Arabic and who can understand English, and questions were presented in both languages in case participants had any difficulty in understanding any questions or wished to check any meanings. Translation and back-translation methods were used to translate the questionnaire from English into Arabic, the authors and a native speaker checked it twice.

The questionnaire included three sections. The **first section** collected data regarding nurses’ personal and professional characteristics, including age, marital status, residential arrangements (i.e., “living with family members”), profession, level of education, work experience, previous training in caring for patients with infectious diseases, and history of attending ethics education programs.

**Section two** included statements that aimed to extract the ethical problems facing nurses in caring for patients in ICU during the MERS-CoV pandemic time. It was developed by Choi and Kim [13], and compromises nine items, with a content validity index of 0.90, and reliability (Cronbach’s α) of 0.83. Nurses were asked to provide their answers with each item on a four-point scale, ranging from 1 (“not at all”) to 4 (“absolutely yes”). It showed good reliability in the current study, with a Cronbach’s α coefficient of 0.89.

The **third section** consists of the Ethical Work Climate Questionnaire as Perceived by Critical Care Nurses, adopted from Cullen and Victor [11]. It includes 36 items distributed over nine dimensions (four items each): self-interest, efficiency, personal morality, organizational profit, friendship, organizational rules and procedures, team interest, laws and professional codes, and social responsibility. Nurses were to respond for each item on a five-point Likert scale, ranging from 0 (“completely false”) to 5 (“completely true”). It has good reliability, with a Cronbach’s α coefficient of 0.83 [11]; in this study, the value was 0.93 for the whole scale. Reversed scoring was applied for negative statements. The total scale score is the sum of all dimensions’ scores (with a possible range of 0–180), whereby higher scores denote more positive perceptions of the ethical work climate by participants.

#### Qualitative component (objective 4)

A qualitative component using written narratives was utilized to enable participants to share as much information as they desired. The objective was to uncover areas that could not be uncovered through the questionnaire and allow deeper understanding of the issues under the study. Nurses who finished the questionnaire were asked concurrently to answer an author-developed three narrative questions to allow them to express their experience with ethical problems more comprehensively and enrich the quantitative data with more illustrative texts. A similar approach was used in earlier studies [23, 24]. The three narrative questions were (1) What kind of ethical problems do you face in your daily work in ICU while caring for patients with infectious diseases/in isolation room? (2) Please share with us the details of an ethical issue which you faced and consider relevant to be reported. (3) Tell us about your response to the ethical problem you faced?

### Data collection

A list of all participants who were involved in direct care of ICU patients with infectious diseases in the selected settings. Recruitment was managed via appointments arranged by the data collector in each country. Participants in each setting who agreed to take part in this study were asked to sign an informed consent form. The questionnaire was provided to them either soft copy or hard copy (in some settings), and it took them 10 min to complete. Data collectors were available in the selected settings to clarify the participants’ quires. The questionnaire was modified by the authors to ask participants about some proposed ethical problems they might face while they are caring for patients with infectious diseases in general (not specifically the MERS-CoV disease). After participants responded to all questionnaire questions, they were asked to answer three-narrative questions. All texts were written down by nurses anonymously and took around 15 min.

### Data analysis

#### Quantitative data analysis

Critical care nurses’ personal and professional characteristics and their associations with ethical work climate and problems were assessed using SPSS software (version 28.0). Cronbach’s alpha was used to test the reliability of the tool. Descriptive statistics with frequencies and percentages or mean and standard deviation (SD) values were used to describe the demographic characteristics of the sample, in addition to the total score of the ethical problems and ethical work climate in the ICU. Non-parametric tests (Kruskal Wallis, Mann Whitney U, and Spearmen correlation coefficient) were used to examine the relationship between ethical work climate and perceived ethical problem statements and nurses’ personal and professional characteristics.

#### Qualitative data analysis

We used content analysis approach [25] to examine the participants’ responses to the three narrative open-ended questions. Each relevant statement was given a code that conveyed its meaning, patterns were found across the transcript, and codes were then compiled into themes. A tree diagram was used to arrange and describe the findings, after multiple rounds of debate led to agreement on the overall conceptual topography of the findings.

### Trustworthiness

The trustworthiness of the qualitative component in this study was assessed using credibility, transferability, dependability, and confirmability [26–28]. Credibility was attained through involving participants of different experiences from varied hospitals in the four studied Arab countries. Experts’ corrective views on the data extraction, analysis, coding, and categorization were considered. To allow reader scrutiny, for transferability, we described the study phases, including the study settings, sampling procedure, and how the data were acquired. To make sure that their intended meaning was conveyed in the transcripts, two ICU nurses were asked to evaluate them.

Dependability and confirmability were emphasized by preparing detailed drafts of the study phases to enable authors to follow the data and its source, as well as comprehend each other’s data interpretations. Also, over the data collection period, the authors had a biweekly conversation to assess the consistency of their perceptions and assessments. The authors were anonymous to all participants in the study guaranteeing reflexivity.

### Ehical considerations

Ethical approval was obtained from the Research Ethics Committee of the Faculty of Nursing, Alexandria University, Egypt (approval number: 2023-9-138); the Institutional Review Board (IRB) at the Applied Science Private University, Jordan (approval number: 2022-2023-2-2); the Institutional Review Board at Princess Nourah bint Abdulrahman University, KSA (approval number: 21–0233); and the MOHP Research Ethics Committee, UAE (approval number: MOHAP/DXB-REC/AMM/No.33 /2021). Written consent, either online or in hard copy, was obtained from each participant before data collection, following explanation of the study details. A large number of participants, however, did not want to narrate their experience.

## Findings

### Quantitative results

#### Participant characteristics

The descriptive statistics for the characteristics of the 635 participating ICU nurses are presented in Table 1. Their mean age was 33.0 ± 8.01 years. Females consisted of 65.7% (*n* = 417) of the total sample. Similar proportions were recruited from each of the four countries, conferring geographical representativeness: 26.8% (*n* = 170) from Egypt, 22.7% (*n* = 144) from Jordan, 25.4% (*n* = 161) from KSA, and 25.2% (*n* = 160) from UAE. Most participants were married (72.1%, *n* = 458) and had children (63.0%, *n* = 400). In terms of professional characteristics, the majority of participants (77.5%) worked as ICU staff, with mean work experience of 10.24 ± 7.41 years. More than half (57.2%) of had a bachelor’s degree. Regarding the ethical issues related to training, more than half of the participants (56.9%, *n* = 361) reported having received adequate ethics training workshops (Table 1).

**Table 1:** Participants’ Characteristics (*n* = 635)

Characteristics	n (%) or Mean ± SD
Personal characteristics	
**Age**	33.0 ± 8.01
**Gender**	
Male	218 (34.3)
Female	417 (65.7)
**Nationality**	
UAE	160 (25.2)
KSA	161 (25.4)
Egypt	170 (26.8)
Jordan	144 (22.7)
**Marital status**	
Single	155 (24.4)
Married	458 (72.1)
Divorced	22 (3.5)
**Have a child**	
No	235 (37.0)
Yes	400 (63.0)
**Living with family**	
No	94 (14.8)
Yes	541 (85.2)
**Professional characteristics**	
**Profession**	
ICU Staff Nurse	492 (77.5)
ICU Nurse manager	143 (22.5)
**Work experience (years)**	10.24 ± 7.41
**Level of education**	
Diploma	127 (20.0)
Bachelor	363 (57.2)
Master	145 (22.8)
**Pandemic previous nursing experience**	
No	244 (38.4)
Yes	391 (61.6)
**Ethical training experience** No	274 (43.1)
Yes	361 (56.9)

UAE, United Arab Emirates; KSA, Kingdom Saudi Arabia; SD, standard deviation

#### Description of study variables

Table 2 represents the frequency of different incidents of ethical problems reported by ICU nurses. According to their report, caring for infected patients was stressful (2.49 ± 1.09), followed by their concerns about assignment to infected patients (2.45 ± 1.03) (maximum score 4). They found less conflict with changing their jobs because of caring for infected patients (1.79 ± 1.02). Table 3 shows the ethical work climate as perceived by ICU nurses. The highest mean score was for the “Organizational rules and procedures” subscale (M = 14.94, SD = 3.50), while the lowest was for the “Self-interest” subscale (M = 10.42, SD = 3.7). For ethical work environment, the mean score was 117.85 of 180 (SD = 24.42). Using percentile calculation, Quartile 4 ranged from [0–45], Quartile 3 ranged from [46–90], Quartile 2 ranged from [91–135], and Quartile 1 ranged from [136–180]. The ICU nurses’ mean perception score toward their ethical work climate fell in Quartile 2.

**Table 2:** Ethical problems in caring for patients with infectious diseases

Variables	Mean ± SD
I worry about caring for an infected patient	2.31 ± 1.05
If I have to choose between infected patients and other kinds of patients, I will care for other kinds of patients.	2.45 ± 1.03
It will be stressful for me to take care of infected patients.	2.49 ± 1.09
If I am not requested by infected patients, I will not provide additional care by myself.	2.06 ± 1.02
It is necessary to reduce holistic care for infected patients.	2.24 ± 1.10
Infected patients should be transferred to a hospital other than my hospital.	1.99 ± 1.07
If I have to take care of infected patients every day, I will quit the job.	1.91 ± 1.11
I would like to change my job because of caring for infected patients.	1.79 ± 1.02
If possible, I would like to move to another ward where I do not have to contact infected patients	1.93 ± 1.11

SD, standard deviation

**Table 3:** Ethical work climate as perceived by ICU nurses

Subscales	Mean ± SD
Self-interest	10.42 ± 3.7
Efficiency	13.90 ± 3.5
Personal morality	10.97 ± 4.24
Organizational profit	12.39 ± 3.45
Friendship	13.51 ± 3.58
Organizational rules and procedures	14.94 ± 3.21
Team interest	13.96 ± 3.46
Laws and professional codes	14.19 ± 3.26
Social responsibility	14.24 ± 3.50
Total score	117.85 ± 24.42

SD, standard deviation

#### Description of study variables

Table 2 represents the frequency of different incidents of ethical problems reported by ICU nurses. According to their report, caring for infected patients was stressful (2.49 ± 1.09), followed by their concerns about assignment to infected patients (2.45 ± 1.03) (maximum score 4). They found less conflict with changing their jobs because of caring for infected patients (1.79 ± 1.02). Table 3 shows the ethical work climate as perceived by ICU nurses. The highest mean score was for the “Organizational rules and procedures” subscale (M = 14.94, SD = 3.50), while the lowest was for the “Self-interest” subscale (M = 10.42, SD = 3.7). For ethical work environment, the mean score was 117.85 of 180 (SD = 24.42). Using percentile calculation, Quartile 4 ranged from [0–45], Quartile 3 ranged from [46–90], Quartile 2 ranged from [91–135], and Quartile 1 ranged from [136–180]. The ICU nurses’ mean perception score toward their ethical work climate fell in Quartile 2.

#### Correlations between study variables

Pairwise correlations between personal and professional characteristics and the total score of ethical work environment are shown in Table 4 perceived ethical work environment showed a significant positive relationship with three personal characteristics, age, gender and number of children, (*p* = 0.002, 0.001, and 0.004, respectively), but not marital status (*p* = 0.516) and living with family members (*p* = 0.208). In terms of professional characteristics, a significant positive relationship was found between perceived ethical work environment and four characteristics (*p* = 0.001, 0.008, 0.004, and 0.001 respectively), but not for nurses’ pandemic previous experience (*p* = 0.094). Moreover, perceived ethical work environment scores between participants in different workplaces differed significantly ( *p* < 0.001), with higher scores among Egyptian ICU nurses as it might be related to that nursing education has started early in Egypt and there are lot of managers and staff with wide experiences. Using Spearman’s correlation coefficient, five ethical problem statements perceived by ICU nurses were not significantly correlated with ethical work environment (*p* = 0.436, 0.216, 0.266, 0.126, and 0.520, respectively), but were for statements 2, 3, 4, and 7 (*p* = 0.000, 0.002, 0.025, and 0.007, respectively).

**Table 4:** Pairwise correlations between personal and professional characteristics and the total score of ethical work environment (*n* = 635)

Personal characteristics	Age ^a^	Gender ^c^	Marital status ^b^	Living with family members ^c^	No. of children ^a^
Total score ethical work environment	0.002*	0.001*	0.516	0.208	0.004*
**Professional characteristics**	**Level of education** ^**b**^	**Profession** ^**c**^	**Pandemic previous nursing experience** ^**c**^	**Previous ethical training** ^**c**^	**Years of experience** ^**a**^
Total score ethical work environment	0.001*	0.008*	0.094	0.004*	0.001*

a Spearmen’s correlations test

b Kruskal- Wallis test

c Mann Whitney test

*The significance level is 0.05

### Qualitative findings

Participants differed in age, gender, and geographic location, providing a wide-ranging, rich sample of experiences to the study. However, not all participants filled out the narrative questions. Only those who provided the answers to the questions were included in the qualitative analysis (*n* = 89). Three themes were identified from the analysis of the resultant data: ethical sense of failure, environment condemnation, and response to ethical conflicts, as presented in Table 5 and described below.

**Table 5:** Themes and units of meaning from the narrative analysis

Themes	Unit of meaning
Ethical sense of failure	Ignorance of autonomy, imbalance between patient care & personal safety [self-demand], delivering bad news [empathy], injustice, inadequate care quality [beneficence]
Environment deficiencies	Insufficient resources including time, incompetent staff, processes for resolving conflicts job demotivation & dissatisfaction, lack of proper communication channels, reporting unethical acts,, policies & regulations
Instant action plan for resolving ethical conflicts	Task management, teamwork, metacognitive strategies [problem-solving], ethical consultation, ethical resilience, organizational support [resources availability], personal coping strategies

#### Ethical sense of failure

Participants reported that they “always” faced ethical problems while they cared for patients with infectious diseases. Despite their varied contexts and features (as discussed previously), most of them agreed on common ethical conflicts that left them emotionally exhausted, and which undermined their performance. They felt guilty for failing to behave ethically in some situations, and to follow ethical principles. A patient or surrogate has the right to participate in care-related decisions according to the principle of autonomy, but some participants reported that they felt unable to follow this principle in their care delivery.

…[It was] difficult to keep the surrogates informed with all decisions especially with the high workload… (P5).…A mother of 19-years girl patient was daily staying outside the ICU team to follow her daughter’s medical condition….either myself or my colleagues found few minutes to update her on her patient. It was really out of hands as we have a large amount of work throughout the shift…(P73).

Another source of suffering was failing to act based on their experiences.

…[I] worked hardly to provide the target quality of care, but [I] couldn’t… (P24). Another participant mentioned that some failure to act situations happened, but unintentionally.

….one time [I] forgot to give the patient a dose of antibiotics. It was only once, however, this was a big mistake because of the multiple responsibilities’ (P523).

They revealed that the financial hardship, workforce shortage, heavy workloads, and (in some cases) a lack of resources contributed to this issue. Moreover, the numbers of bad news they communicated to the families either due to deterioration or death, which sapped their personal morale.

…The situations [we] faced were beyond our empathic capacities, [we] really experienced a cocktail of emotions, for example, emotional fatigue, guilt, and moral injury… (P323).

Besides, they linked their suffering to unfair distribution between the staff working in the isolation units.

…[I] felt guilty to refuse a duty, therefore, [I] thought in resignation… (P246).

The participants’ narratives also reflected that they perceived imbalance between continuation of patient care and their personal safety (risk for cross-infection).

…[I] felt as [I am] in a dilemma…. should [I] continue caring for infected patient despite the high risk to infect myself or my family or renege from patient’s care… (P89).

#### Environment deficiencies

Despite data being collected from varied participants who are working in different environments, most participants cited resource limitations as a common feature. In some units this was manifest in the form of equipment, while in others it was evident in limited time and high workload, unsafe staff ratios, and incompetent staff.

…Staff [ICU nurses] who are working here have a high patient ratio, with hyper-dynamic task cycles… (P498).

Most participants related their inability to adapt to the ethical climate because of job demotivation and dissatisfaction, especially for those who spent long shifts providing healthcare service in ICU isolation units.

…[We] need a motivation to continue working in such a drained environment, for example, rewards, promotions, or remuneration… (P12).

….[I] am personally afraid of getting infected while caring for patients. I have three kids, and I feel anxious about the possibility of getting infected and the impact it could have on my family…(P 344).Moreover, participants added that the processes for resolving conflicts might lead to unresolved tensions and dissatisfaction among them.

…The workflow, how difficult situations are usually managed, and for how long these conflicts exist always affect my satisfaction, well-being and acts (P159).

Further, lack of proper communication channels leading to misunderstandings, confusion, or misinformation.

…I believe that clear and honest communications between us as nurses and the top management would increase our loyalty and morale. The style of communication can reinforce the one’s attitudes and behaviors (P10).

Few participants showed concerns on reporting unethical practices or violations to their supervisor.

…It’s not granted if I told my supervisors, they would understand the situation…[I] got scared of blame, and termination (P23).

They suggested that policy makers develop policies and regulations to regulate their working schedule in the isolated ICUs.

…[I] perceive that we need a policy that regulate our assignments to infected patients… (P35).

#### Instant action plan for resolving ethical conflicts

Most participants suggested that teamwork could support their responses to these ethical challenges and support either from managers or peers.

…[I] usually share ideas and suggestion with my colleagues…[I] feel this would help [me] doing the best for patient… (P45).

…task management through prioritizing tasks could decrease the ethical conflicts [we] faced in the everyday practice… (P112).

Participants also recommended continuous ethical training to improve ethical resilience, consultation, and decision making.

…[We] need continuous training to guide us how to manage different ethical situations… (P189).

…[I] need to read different scenarios as much as [I] can, so these could guide [me] how to act ethically (P18).

Also, enhancing metacognitive strategies to reinforce problem-solving and decision-making skills was recommended by multiple nurses.

…ICU nurse mangers should schedule [us] for a serious of workshops that allow recognizing the ethical conflicts, critically analyze the situations, and offering possible solutions while maximizing the use of the available resources… (P55).

Participants believed that the ethical climate/environment has an important role in their experience and response to ethical conflicts. Based on their narrations, the environment covers two key aspects: organizational support, resource availability, and personal coping strategies.

…[I] believe that the organization should encourage our endeavors…for example, training, motivation, rewards… (P93).

…resources [equipment, policies, and consultancy services] supplement our practical, problem solving, ethical skills… (P1).

…[We] should adopt certain coping strategies to help in handling the different situation in the everyday environment, for example, balancing the work-life activities, having emotional support, engaging into related training (P89).

## Discussion

The present study identified the perceived ethical work climate and problems among nurses in adult ICUs caring for patients with infectious diseases across a sample of four Arab countries. The findings showed the overall ethical work climate falls in quartile 2, and that the ethical work climate is significantly associated with ICU nurses’ personal and professional characteristics such as age, gender, number of children, level of education, profession, previous ethical training and years of experience. The qualitative findings highlighted that the ethical work climate played a role in ICU nurses’ experience of the various daily ethical conflicts. The qualitative findings disclosed more details of ICU nurses’ experiences with ethical problems and how they would respond to these existing ethical conflicts. ICU managers, clinicians, and policymakers should consider the recommended ethical strategies to target ICU nurses who are usually facing similar conflicts since despite over half of them had ethical training, they are requesting more support.

Prior studies concluded that ICU nurses commonly face some ethical problems while caring for patients with infectious diseases [13, 29, 30], similar to the findings of the current study. While the statements used in the questionnaire did not cover all ethical problems encountered in ICUs, the qualitative component of the study provided additional insight into other ethical issues and conflicts.

The scores of all the means of proposed ethical problems statements had a minimal difference between the lowest and the highest values, suggesting that participants’ experiences are, to some extent, similar across the different statements. This was in line with a study conducted in Korea [13]. Similar findings have been reported by other studies that utilized different methods to identify ethical conflicts [4, 31].

In terms of the ethical work climate, most ICU nurses perceived that organizational rules have a contribution to nurses’ perception to the ethical work environment, similar to a study conducted by Dalmolin et al. [32]. This could be due to the ethical work environment attributed to the organizational culture, where bedside nurses are involved in shared decision-making [33]. Few of them perceived that self-interest would impact the ethical work environment, contrary to the findings of Sheedy et al. [34], which indicated that low ethical egoism enables the risk climate to exert a more significant influence on unethical pro-organizational behaviors.

In an independence climate, workers are expected to be guided by their personal beliefs [35]. Accordingly, the current findings showed that the ethical work climate is significantly associated with participants’ age, gender, and workplace. An ethical work climate is ascertained from workers’ general observations and opinions of the organization, rather than their individual attitudes and thoughts regarding how ethical problems are addressed and possible solutions [36]. In accordance with this definition, the findings also showed that nurses’ level of education, job rank, years of experience, and previous training were significantly associated with their positive perception to the ethical work climate. Participants’ narrations also linked the lack of training and incompetent staff with negative work climate perceptions. This is consistent with Okumoto et al. [37], who documented that the ethical climate of nurses in three Japanese teaching hospitals showed a significant association with hospital, gender, unit specialty, experience of ethics education, in-service ethical training, and workshops/ academic conferences on nursing ethics. In their narrations, participants suggested the policymakers to create specific policies targeting the isolation ICUs. They also asked the educators for continuous ethical training and emphasized the importance of ICU managers encouraging teamwork spirit.

Moreover, the quantitative results highlighted that the ethical work climate was significantly associated with some ethical problems, and these results were affirmed qualitatively, suggesting that the different healthcare systems targeting these ethical problems, while concurrently leverage the available resources, equipment, and workforce. According to the results of this study, the most common ethical problem experienced by ICU nurses while caring for patients with infectious diseases was having a mind-set of patient avoidance. The items showing the mind-set issue were as follows, in descending order of score: “It will be stressful for me to take care of patients with infectious disease”; “If I have to choose between infected patients and other kinds of patients, I will care for other kinds of patients”. Personal and family safety issues, fear of getting infected and infecting their families, afraid of abiding by non-maleficence, failure to act, urgent self-demands, and ignored autonomy as reported in their narrations might explain this avoidance.

The results of previous studies suggest the need for a positive ethical climate to support and help ICU nurses stay committed to delivering high-quality patient care while struggling with infectious diseases [9, 34, 38, 39]. Our results showed an association between ethical work environment and related ethical problems, which also supports this suggestion. To our knowledge, ethical problems and their association with ethical work environment was not studied in previous studies; instead, most researchers focused on job satisfaction [9], and ethical sensitivity and quality of care [38]. Additionally, participants’ narrations disclosed their concerns about insufficient resources (either in terms of equipment or incompetent staff), the process of resolving conflicts, proper communication challenges, reporting unethical acts and job satisfaction, which interfered with their ability to deal with everyday ethical problems. These findings affirm those of prior studies [9, 33, 36].

### Strengths and limitations

The use of a mixed-method design in this study helps in achieving a deeper understanding of the association between the ethical work climate and proposed ethical problems in four low- and medium-income Arab countries. The results of the current study encourage future research studying predictors of the ethical work climate perceptions among ICU nurses. However, a number of limitations merit mentioned. First, convenience sampling might make it harder to generalize our results. Second, the data were collected only from ICU nurses, without considering other healthcare providers. Third, potential social desirability bias, given that the instrument implicitly queries nurses about their own personal and professional ethics. Fourth, using an online survey rather than face-to-face questionnaire.

## Conclusions

This study targeted ICU nurses in Arab countries who directly cared for patients with infectious diseases. The findings provide insights into the association between the ethical climate and ICU nurses’ personal and professional characteristics including cultural background, in addition to their perceptions of ethical problems, based on using a mixed-method approach. The results indicated that ICU nurses had a relatively fair perception of the ethical work climate, with implications for addressing ethical issues and conflicts in various settings. Further, the study shed the light on the ethical and environmental deficiencies while suggesting instant actions to be followed by the ICU nurses, mangers, and policy makers. Further research is needed to determine predictors of ethical work climate perceptions among ICU nurses with regard to the differences between Arabic and non-Arabic speakers.

### Electronic supplementary material

Below is the link to the electronic supplementary material.

Supplementary Material 1

Supplementary Material 2

## Data Availability

All data generated or analysed during the study are available from the corresponding author [Mohannad Anuruz] on request.

## Abbreviations

ICUs: Intensive care units
KSA: Kingdom of Saudi Arabia
UAE: United Arab Emirates
